# The Effect of Federal Policy Changes on Buprenorphine Prescribing in Massachusetts

**DOI:** 10.1016/j.acepjo.2025.100246

**Published:** 2025-09-09

**Authors:** Jane M. Hayes, Rebecca E. Cash, Neha Jia Ahmad, Julia Menzies, Danielle DeNufrio Valerio, Netrali Dalvi, Leonard D. Young, Scott G. Weiner

**Affiliations:** 1 Harvard Affiliated Emergency Medicine Residency at Mass General Brigham, Boston, Massachusetts, USA; 2 Department of Emergency Medicine, Massachusetts General Hospital, Boston, Massachusetts, USA; 3 Massachusetts Department of Public Health, Boston, Massachusetts, USA; 4 Department of Emergency Medicine, Brigham and Women’s Hospital, Boston, Massachusetts, USA

## Abstract

**Objectives:**

Two federal policy changes were recently implemented to expand access to buprenorphine. Our objective was to determine whether these policies were associated with a change in the trend of buprenorphine prescribing in Massachusetts, specifically among specialists including emergency physicians (EPs).

**Methods:**

The Massachusetts Prescription Drug Monitoring Program database was searched for buprenorphine prescription records between May 2020 and January 2024. Monthly counts of buprenorphine prescriptions were measured for 12-month pretime and posttime periods for each policy. Monthly prescriptions were stratified by specialties of interest, and interrupted time series analyses evaluated the impact.

**Results:**

There was no change in the total buprenorphine prescriptions per month (bppm) between the prepractice-guideline period compared with the postpractice-guideline period nor between the pre-no-X-waiver period compared with the post-no-X-waiver period in Massachusetts. Among EPs, there was no change in prepractice guideline (−9.9 bppm, 95% CI, −33.4 to 13.7) and a declining trend in the postpractice-guideline of 15.6 (95% CI, −27.9 to −3.4) fewer bppm. Among EPs, there was a declining trend of 17.8 (95% CI, −26.9 to −8.6) fewer bppm in the pre-no-X-waiver period, which flattened (5.7 bppm, 95% CI, −18.7 to 7.3) in the post-no-X-waiver period.

**Conclusion:**

The analysis demonstrates that the 2 federal policy changes were not associated with a significant change in the trend of total buprenorphine prescriptions written monthly in Massachusetts. The magnitude of decline in monthly buprenorphine prescriptions among ED physicians may have been tempered by the elimination of the X-waiver.

**The Bottom Line**

We investigated the impact of 2 recent federal policy changes—the implementation of the 2021 US Department of Health and Human Services guideline and the elimination of the X-waiver in 2023—on buprenorphine prescribing in Massachusetts. There was no significant change in the trend of monthly buprenorphine prescriptions overall. The elimination of the X-waiver may have tempered the declining trend in buprenorphine prescriptions written by emergency physicians. This study highlights that federal deregulation of buprenorphine was not sufficient to expand prescribing of buprenorphine treatment.

## 1 Introduction

### 1.1 Background

In the United States, the number of deaths attributed to opioids was on an alarming upward trajectory, increasing 6-fold from 1999 to 2021.^1^ Buprenorphine, a synthetic partial opioid agonist, is a medication approved for opioid use disorder (OUD) treatment.^2^ Buprenorphine decreases opioid use by blocking the effect of full agonists while also reducing cravings and withdrawal symptoms. Despite evidence for the effectiveness of medications for opioid use disorder (MOUD, including buprenorphine, methadone, and naltrexone),^3^, ^4^, ^5^, ^6^, ^7^ most individuals with OUD do not receive MOUD treatment.^8^ Prior work estimated that only 25% of individuals in need of OUD treatment received medication in 2022.^9^

The historical requirement for clinicians to obtain an X-waiver from the Drug Enforcement Administration (DEA) to prescribe buprenorphine has been cited as a potential barrier to its wider use in the United States.^10^^,^^11^ To acquire an X-waiver, physicians were previously required to complete an additional 8 hours of training. Advanced practice clinicians (APCs) were permitted to prescribe in 2016 as long as they also completed an additional 24 hours of training.^12^^,^^13^ After completing the training, there were additional regulatory hurdles that clinicians with X-waivers faced such as increased DEA scrutiny with periodic audits.^11^ The administrative barriers to obtaining an X-waiver may have contributed to the historically modest number of buprenorphine-prescribing clinicians,^14^^,^^15^ with estimates that <5% of licensed physicians were credentialed to prescribe buprenorphine in 2020.^16^ Two federal policy changes were implemented, with the intent to expand access to buprenorphine for OUD treatment.

First, the US Department of Health and Human Services (HHS) released its Practice Guidelines for the Administration of Buprenorphine for Treating OUD in 2021.^17^ This allowed clinicians to prescribe buprenorphine to a maximum of 30 patients simultaneously, without additional training.^17^ This change was implemented to encourage less frequent prescribers, such as emergency physicians (EPs) and primary care physicians, to prescribe buprenorphine.

Second, fewer than 2 years later, the Consolidated Appropriations Act of 2023 completely removed the X-waiver federal requirement and all clinicians with Schedule III authority can now prescribe buprenorphine without patient limits or any specific education.^18^^,^^19^ However, to renew their license, all DEA registrants must complete 8 hours of training on the treatment and management of patients with opioid or other substance use disorders or attest to having specialty training in addiction medicine, even if they do not prescribe buprenorphine. This policy change allows prescribing of buprenorphine without a patient cap.

### 1.2 Importance

Although these 2 policy changes removed barriers to prescribing buprenorphine, it is unclear how many prescribers are using this new authority. After buprenorphine was deregulated in France, there was a 79% decline in overdose deaths from 1995 to 1999.^20^ During the same timeframe, MOUD treatment increased by >95%.^20^ A prior study theorized that if this success is extrapolated to the United States, there would be a decline of >30,000 overdose deaths per year.^11^

### 1.3 Goals of This Investigation

A critical first step to determine the impact of buprenorphine deregulation in the United States is to evaluate if these recent policy changes are associated with shifts in buprenorphine prescribing. Our objective was to determine if these 2 federal policy changes aimed at expanding access to buprenorphine for OUD treatment were associated with a change in the trend of buprenorphine prescriptions in the state of Massachusetts in general and specifically among specialties of interest, including emergency medicine.

## 2 Methods

### 2.1 Study Design, Setting, and Data Source

We conducted an interrupted time series analysis of monthly buprenorphine prescriptions in Massachusetts between May 1, 2020, and January 31, 2024, to evaluate the impact of the 2 federal policy changes.

The study timeframe includes 1 year before the first policy change was implemented and 1 year after the second policy change became effective. Deidentified data were extracted from the Massachusetts Prescription Drug Monitoring Program (PDMP). This database contains prescription records for federally controlled medications, including buprenorphine prescriptions, reported by retail pharmacies in the state of Massachusetts as well as mail order pharmacies dispensing to Massachusetts residents. This study was deemed not to constitute human subjects research by the Mass General Brigham Institutional Review Board. This study followed the Strengthening the Reporting of Observational Studies in Epidemiology reporting guideline.^21^

### 2.2 Measurements

From the Massachusetts PDMP, we extracted data on the name of the prescribed drug, the National Drug Code (NDC) to determine the formulation of the prescribed drug, age of patient, sex of patient, month and year that the prescription was written, the professional degree of the prescriber, and the prescriber’s specialty(ies).^22^ We classified clinicians with a professional degree of Doctor of Medicine (MD) or Doctor of Osteopathic Medicine (DO) as physicians. We classified clinicians with a professional degree, role, or specialty level of physician assistant or nurse practitioner as APCs. NDCs were used to identify prescriptions of buprenorphine formulations that are typically used for OUD treatment, as opposed to pain management, dispensed in Massachusetts (see Supplementary Appendix 1).

Monthly statewide total buprenorphine prescriptions as recorded by the Massachusetts PDMP were measured during 12-month pretime and posttime periods for the 2 policies studied in this investigation. The first policy intervention of interest was the HHS guideline that allowed clinicians to prescribe buprenorphine for up to 30 patients without X-waiver training.^17^ This policy became effective April 28, 2021. The second policy intervention of interest was the law that was introduced with the Consolidated Appropriations Act of 2023, which completely removed the X-waiver and allowed clinicians to prescribe buprenorphine without a patient cap.^19^ This policy became effective January 12, 2023. There were no new state-specific regulations pertinent to buprenorphine enacted during these periods.

### 2.3 Outcomes

The primary outcome of interest was the trend in statewide total buprenorphine prescriptions per month (bppm) by all clinicians across the state of Massachusetts in the 12-month pretime and posttime periods for both federal policy changes. Secondary outcomes include the trend in statewide bppm written by physicians in reported specialties of interest including emergency medicine, combined internal medicine (IM) and primary care, psychiatry, and pediatrics during the same time periods. We also evaluated the trend in total bppm written by APCs across all specialties during the same time periods. We did not evaluate APCs based on specialty as their primary specialty can be more fluid, and thus, there was a greater chance of misclassification if their current specialty was not updated in the PDMP.

### 2.4 Analysis

We used descriptive statistics to report patient characteristics in large aggregates prepolicy and postpolicy changes. We also stratified prescriptions by both the clinician’s professional degree and specialty. To understand the immediate and longer-term change over time on statewide monthly buprenorphine prescriptions, we used separate interrupted time series analyses with each of the 2 policy changes as the interruptions. For the practice guideline analysis, we compared monthly buprenorphine prescribing from May 2020 to April 2021 (prepractice guideline) vs from May 2021 to April 2022 (postpractice guideline). For the X-waiver analysis, we compared monthly buprenorphine prescribing from February 2022 to January 2023 (pre-no-X-waiver) to February 2023 to January 2024 (post-no-X-waiver). We repeated the same time series analyses described above stratified by the 5 cohorts of interest: EPs, IM/primary care physicians, psychiatrists, pediatricians, and APCs. There was a small amount of missingness: approximately 2% of buprenorphine prescriptions were missing either the prescriber’s professional degree, role, or specialty. Buprenorphine prescriptions missing these variables were included in the total calculations but were excluded from analyses stratified by prescriber type. No hypothesis testing was performed as the large sample size could result in a statistically significant result in the absence of clinical relevance. We instead report confidence intervals to illustrate the magnitude of the effect of the analysis. Analysis was conducted using Stata IC 15.1 (StataCorp).

## 3 Results

During the study period, there were 2,826,824 total buprenorphine prescriptions. The median length of prescription (ie, days’ supply) was 14 days (IQR, 7-28). Most patients who received a buprenorphine prescription were male (60.9%) with a median age of 42 years (IQR, 35-53 years).

### 3.1 Total Buprenorphine Prescriptions Prepractice and Postpractice Guideline

**Figure 1:**
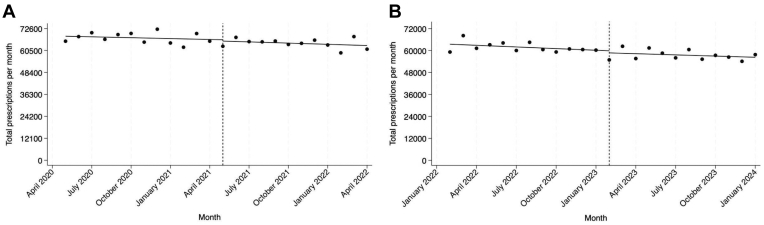
Interrupted time series analysis of total buprenorphine prescriptions in Massachusetts. A, Health and Human Services guideline implemented. B, Buprenorphine X-waiver eliminated.

There were 809,700 and 773,573 total buprenorphine prescriptions written in the prepractice- and postpractice-guideline periods, respectively (Table 1). In the prepractice-guideline period, there was no change in the number of monthly buprenorphine prescriptions (−162.5 bppm, 95% CI, −555.3 to 230.3, Table 2, Fig 1). There was no level change in the month following the implementation of the HHS practice guideline (−727.3 bppm, 95% CI, −4254.4 to 2799.8). In the postpractice-guideline period, there remained no change in the number of monthly buprenorphine prescriptions (−233.1 bppm, 95% CI, −570.6 to 124.4). These findings indicate no change in buprenorphine prescribing associated with the practice guidelines policy change.

**Table 1:** Characteristics of buprenorphine prescriptions and patients receiving these prescriptions in Massachusetts, May 2020 to January 2024.

Variable	Prepractice guideline May 2020-April 2021	Postpractice guideline May 2021-April 2022	Pre-no-X-waiver February 2022-Jan 2023	Post-no-X-waiver February 2023-Jan 2024
Total buprenorphine prescriptions	809,700	773,573	741,824	690,311
Median (IQR) length of prescription, days	14 (7-28)	14 (7-28)	14 (7-28)	15 (7-28)
Patient characteristics				
Male patients (%)	61.0	61.0	60.9	60.8
Median (IQR) age, y	41 (34-51)	42 (35-52)	43 (36-53)	44 (37-54)
Patients per mo, median (IQR)	38,286.5 (37,947.0-38,499.5)	37,485.5 (37,400.5-37,583.5)	37,014.0 (36,672.5-37,274.5)	35,745.0 (35,523.0-36,151.0)
Prescribers per mo, median (IQR)	2611.5 (2398.5-2877.5)	2522.0 (2504.0-2554.0)	2668 (2645.0-2693.5)	2872.5 (2744.5-2896)

**Table 2:** Interrupted time series regression analysis of total buprenorphine prescriptions in Massachusetts prepractice guideline and postpractice guideline.

Variable	Absolute change in total buprenorphine prescriptions per mo β (95% CI)
Base trend in prepractice-guideline segment	−162.5 (95% CI, −555.3, 230.3)
Change in level in the postguideline segment	−727.3 (95% CI, −4254.4, 2799.8)
Change in trend (slope) in the postguideline segment	−60.6 (95% CI, −604.3, 483.2)

### 3.2 Total Buprenorphine Prescriptions Pre- and Post-no-X-waiver

There were 741,824 and 690,311 total buprenorphine prescriptions written in the pre- and post-no-X-waiver periods, respectively. In the pre-no-X-waiver period, there was no change in the number of monthly buprenorphine prescriptions (−300.0 bppm, 95% CI, −645.0 to 44.9, Table 3). There was no level change in the month following the elimination of the X-waiver (−1144.4 bppm, 95% CI, −4083.7 to 1794.9). In the post-no-X-waiver period, there remained no change in the number of monthly buprenorphine prescriptions (−217.9 bppm, 95% CI, −545.6 to 109.9). These findings indicate no change in buprenorphine prescribing associated with the removal of the X-waiver.

**Table 3:** Interrupted time series regression analysis of total buprenorphine prescriptions in Massachusetts pre-no-X-waiver and post-no-X-waiver.

Variable	Absolute change in total buprenorphine prescriptions per mo β (95% CI)
Base trend in pre-no-X-waiver segment	−300.0 (95% CI, −645.0, 44.9)
Change in level in the post-no-X-waiver segment	−1144.4 (95% CI, −4083.7.0, 1794.9)
Change in trend in the post-no-X-waiver segment	82.2 (95% CI, −391.8, 556.1)

## 3.3 Buprenorphine Prescriptions Prepractice and Postpractice Guideline Among EPs

**Figure 2:**
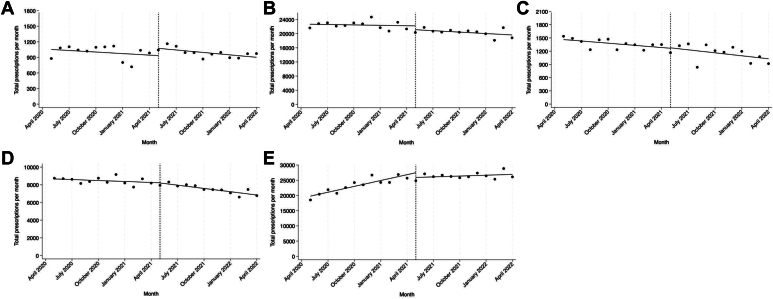
Interrupted time series analysis of buprenorphine prescriptions prepractice and postpractice guideline implementation by specialty. A, Emergency physicians. B, Internal and family medicine physicians. C, Pediatricians. D, Psychiatrists. E, Advanced practice clinicians from all specialties.

The number of buprenorphine prescriptions written by the selected professional cohorts for each time period is reported in Table 4. Among EPs, there was no change in the number of monthly buprenorphine prescriptions in the prepractice-guideline period (−9.9 bppm, 95% CI, −33.4 to 13.7, Fig 2). There was no level change in the month following the implementation of the HHS practice guideline (139.8 bppm, 95% CI, −67.6 to 347.6). However, in the postpractice-guideline period, there was a decline of 15.6 (95% CI, −27.9 to −3.4) fewer bppm. These findings indicate that the practice guideline was associated with a decrease in the number of prescriptions written by EPs.

**Table 4:** Buprenorphine prescriptions by selected specialty cohorts in Massachusetts, May 2020 to January 2024.

Variable	Prepractice guideline May 2020-April 2021	Postpractice guideline May 2021-April 2022	Percent change (%)	Pre-no-X-waiver February 2022-Jan 2023	Post-no-X-waiver February 2023-Jan 2024	Percent change
Total buprenorphine prescriptions						
By professional cohort						
EPs	11,977	11,852	−1.0	10,144	7037	−30.6
IM/family practice physicians	269,477	244,528	−9.3	226,962	208,175	−8.3
Psychiatrists	101,543	90,285	−11.1	78,035	65,177	−16.5
Pediatricians	16,463	13,808	−16.1	11,819	6519	−44.8
APCs from all specialties	279,753	317,177	13.4	324,890	321,247	−1.1
Median (IQR) length of prescription, days						
By professional cohort						
EPs	14 (7-28)	17 (7-28)		21 (7-28)	28 (7-28)	
IM/family practice physicians	14 (7-28)	18 (8-28)		21 (10-28)	28 (12-28)	
Psychiatrists	28 (14-30)	28 (14-30)		28 (14-30)	28 (14-30)	
Pediatricians	14 (7-14)	14 (7-14)		14 (7-16)	14 (7-28)	
APCs from all specialties	12 (7-21)	14 (7-28)		14 (7-28)	14 (7-28)	

APC, advanced practice clinician; EP: emergency physician; IM, internal medicine.

### 3.4 Buprenorphine Prescriptions Pre- and Post-no-X-waiver Among EPs

**Figure 3:**
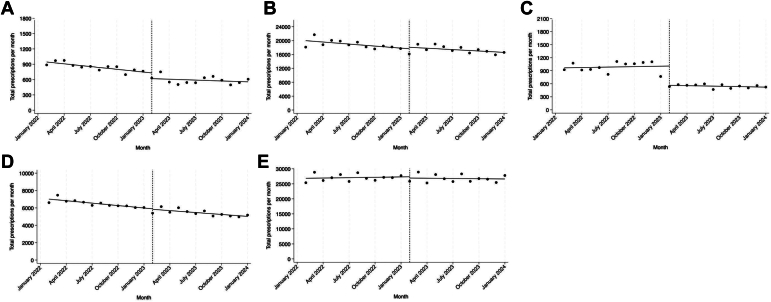
Interrupted time series analysis of buprenorphine prescriptions pre- and post-no-X-waiver elimination by specialty. A, Emergency physicians. B, Internal and family medicine physicians. C, Pediatricians. D, Psychiatrists. E, Advanced practice clinicians from all specialties.

Among EPs, there was a decline of 17.8 (95% CI, −26.9 to −8.6, Fig 3) fewer monthly buprenorphine prescriptions in the pre-no-X-waiver period. There was no level change in the month following the elimination of the X-waiver (−112.0 bppm, 95% CI, −230.6 to 6.6). However, in the post-no-X-waiver period, the monthly declining trend of fewer buprenorphine prescriptions written per month was no longer statistically significant (5.7 bppm, 95% CI, −18.7 to 7.3). These findings indicate that the elimination of the X-waiver blunted the previous decreasing trend of fewer buprenorphine prescriptions per month, but that there was no increase in prescriptions seen.

### 3.5 Buprenorphine Prescriptions Prepractice and Postpractice Guideline Among Other Selected Specialties

IM and primary care physicians experienced no change in monthly buprenorphine prescriptions in the prepractice-guideline period (−39.1 bppm, 95% CI, −219.0 to 140.8) and continued to experience no change in monthly buprenorphine prescriptions in the postpractice-guideline period (−135.0 bppm, 95% CI, −341.8 to 71.9). Pediatricians experienced a declining trend of 16.4 (95% CI, −29.9 to −2.9) fewer monthly buprenorphine prescriptions in the prepractice-guideline period. Pediatricians experienced no change (−22.6 bppm, 95% CI, −47.3 to 2.2) of monthly buprenorphine prescriptions in the postpractice-guideline period, indicating a blunting of the previous declining trend. Psychiatrists experienced no change in monthly buprenorphine prescriptions in the prepractice-guideline period (−35.8 bppm, 95% CI, −86.7 to 15.1) and a declining trend of 123.5 (95% CI, −178.3 to −68.8) fewer monthly buprenorphine prescriptions in the postpractice-guideline period. The APC cohort experienced an increasing trend of 641.0 (95% CI, 447.2 to 834.7) more monthly buprenorphine prescriptions in the prepractice-guideline period. In the postpractice-guideline period, the APC cohort experienced no change (92.3 bppm, 95% CI, −125.9 to 310.5) in monthly buprenorphine prescriptions.

### 3.6 Buprenorphine Prescriptions Pre- and Post-no-X-waiver Among Other Selected Specialties

IM and primary care physicians experienced a declining trend of 194.6 (95% CI, −338.7 to −50.6) fewer monthly buprenorphine prescriptions in the pre-no-X-waiver period and no change (−133.3, 95% CI, −297.6 to 31.1) in the post-no-X-waiver period. Pediatricians experienced no change (3.5 bppm, 95% CI, −21.3 to 28.2) in the pre-no-X-waiver period, but there was a negative level change in the month following the elimination of the X-waiver (−443.6 bppm, 95% CI, −656.3 to −230.9). Pediatricians continued to experience no change (−7.2, 95% CI, −32.4 to 18.0) in the post-no-X-waiver period. Psychiatrists experienced a declining trend of 93.2 (95% CI, −129.8 to −56.6) fewer monthly buprenorphine prescriptions in the pre-no-X-waiver period and continued to experience a declining trend of 74.0 (95% CI, −111.6 to −36.6) fewer monthly buprenorphine prescriptions in the post-no-X-waiver period. The APC cohort experienced no change in monthly buprenorphine prescriptions (42.0 bppm, 95% CI, −165.8 to 249.9) in the pre-no-X-waiver period. In the post-no-X-waiver period, the APC cohort also experienced no change (−27.1 bppm, 95% CI, −270.5 to 216.2) in monthly buprenorphine prescriptions.

## 4 Limitations

Although we were able to evaluate the total number of buprenorphine prescriptions that patients received in Massachusetts, we did not evaluate the number of individual patients who received a buprenorphine prescription nor the number of individual clinicians who prescribed buprenorphine. We are therefore unable to determine the proportion of eligible clinicians who prescribed buprenorphine after the 2 federal policy changes studied in this investigation, or if the prescription was for treatment initiation or continuation. We are also limited by potential information bias, including misclassification. Several variables in the PDMP dataset, such as clinician professional degree and specialty, are self-reported and may contain errors or not be updated to reflect the current practice. It is possible that EPs may have transitioned to other specialties, like a primary practice in addiction medicine, which would not be detected. Prescriptions missing professional degree, role, or specialty variables (2.0%) were excluded from analysis based on clinician characteristics, which may bias our results, especially if there was differential missingness for specific clinician types (ie, MD vs APC) or specialties. We did not differentiate long-acting (ie, extended-release) buprenorphine prescriptions, which may have changed over the study period. We also cannot determine if patients were using the ED to initiate buprenorphine or as a stopgap when they are not able to access their MOUD specialist. The interrupted time series analysis assumes an immediate change following the month that the policy is enacted; however, translation of policy change may take a longer period of time. Results may reflect a change in the prevalence of individuals seeking MOUD treatment, which we could not determine from our data. As we only studied Massachusetts, our findings may not be generalizable to other states. Finally, the associations we found may not identify causality.

## 5 Discussion

In this interrupted time series analysis, 2 recent policy changes, namely the implementation of the HHS practice guideline and the elimination of the X-waiver, did not have the intended magnitude of positive impact on buprenorphine prescribing in Massachusetts. Overall, there was no change in the monthly trend of total buprenorphine prescriptions after either the implementation of the HHS guideline or the elimination of the X-waiver. Among EPs, the implementation of the HHS guideline was actually associated with a declining trend in monthly buprenorphine prescriptions. The elimination of the X-waiver was associated with a blunting of the previously declining trend of monthly buprenorphine prescriptions among EPs, but not the increased trend as hypothesized.

Our findings regarding buprenorphine prescribing across all clinician specialties are similar to previous studies, which found that implementation of the HHS guideline and elimination of the X-waiver did not significantly increase total buprenorphine use. A recent study found that the implementation of the HHS guideline was not consistently associated with an increase in the number of patients receiving buprenorphine.^23^ The authors analyzed data from the HEALing Communities Study for 4 states selected in part for their high burden of opioid deaths (Kentucky, Massachusetts, New York, and Ohio).^23^ The authors found that Massachusetts was the only state where more patients received buprenorphine prescriptions after the implementation of the guideline, whereas there were no significant changes among the other states.^23^ Although we did not analyze the number of individual patients who received a buprenorphine prescription, our study found that the number of monthly buprenorphine prescriptions did not change in association with this policy change in Massachusetts.

A study of buprenorphine prescriptions dispensed from 92% of national retail pharmacies found that the number of buprenorphine prescribers increased, but the total number of patients using buprenorphine did not meaningfully change in the 12 months after the elimination of the X-waiver.^24^ Another study of commercial insurance and Medicare Advantage enrollees similarly concluded that there were no significant increases in buprenorphine prescriptions following the changes in training requirements or removal of the X-waiver.^25^ These studies and the present study suggest that eliminating the additional federal training requirement to prescribe buprenorphine is not enough to meaningfully increase the trend in buprenorphine prescriptions, including prescriptions written by EPs. This is unfortunate, given that 1 in 3 patients receiving a buprenorphine prescription by emergency medicine prescribers goes on to receive a second prescription.^26^ Although disappointing, these results are not entirely surprising. Prior to the elimination of the X-waiver, only half of waivered clinicians regularly prescribed buprenorphine.^27^

The reason for low buprenorphine prescribing is likely multifactorial. Despite having a good safety profile, the X-waiver implied that buprenorphine was a dangerous or more challenging medication to prescribe.^13^^,^^28^^,^^29^ The stigmatization of this medication may have had a chilling effect on both potential prescribers and patients, which will take time and effort to overcome.^30^ Prescribers of buprenorphine historically faced heightened federal scrutiny, including audits by the DEA, which were a time-consuming and daunting process.^11^ Prescribers are frequently required to report these audits to credentialing agencies regardless of the outcome, which can discourage potential prescribers. Unfortunately, patients who use buprenorphine also face increased stigmatization from the public and even healthcare professionals,^31^^,^^32^ which may hinder their desire to pursue buprenorphine treatment. This stigma may rise from the general misconception that buprenorphine substitutes 1 drug for another without understanding its effectiveness in treating OUD.^4^^,^^33^ Physicians cite other barriers to increased buprenorphine prescribing including concerns about lack of professional support, lack of expertise in addiction treatment, and difficulty accessing reimbursement for treatment.^13^^,^^34^ Several states continue to require training despite the elimination of the federal requirement, which serves as another barrier to increased prescribing.^35^ It has become clear that adjusting regulations around buprenorphine is not sufficient to bring about change on its own. Our findings suggest that additional promulgation and education are needed to achieve the translation from policy to meaningful clinical outcomes. Change management could increase training and integrate buprenorphine use into routine practice, thus removing remaining barriers.^36^ In general, practice change after new guidelines or regulatory changes takes time,^37^ and it may have been too soon in this study to detect longer-term differences resulting from these new policies.

Our study period included the COVID-19 pandemic, which was associated with many public health implications. In the midst of the pandemic, Massachusetts experienced a record peak in opioid deaths in 2021, which was only surpassed in 2022 with an opioid–related overdose death rate of 33.5 per 100,000 people.^38^ These data illustrate that the prevalence of opioid use was likely increasing during the pandemic with a heightened need for access to MOUD. The X-waver was eliminated at the beginning of 2023, which is the same year the pandemic officially ended.^39^ Fortunately, this same year in 2023, we saw a decline in opioid deaths by 10% in the state.^40^ It is possible that the prevalence of opioid use was lower in 2023, which might explain the modest change in the number of buprenorphine prescriptions written. However, the decreased opioid deaths may also be related to a larger public health initiative in the state to increase fentanyl test strip and naloxone availability. The pandemic also created a dynamic landscape of patients’ ability to access healthcare. It was very challenging to be seen in an outpatient physical location during the pandemic, and we expected to see a larger increase in the reliance to obtain buprenorphine prescriptions in the ED during this period. As we do not see this increase, it is possible that patients were receiving buprenorphine prescriptions from telemedicine visits as the use of telemedicine increased from 15.4% prior to the pandemic to 86.5% in 2021.^41^

Although we were unable to evaluate prescribing trends for emergency medicine APCs specifically, we were able to evaluate trends among all APCs in Massachusetts and the findings were different from the physician cohorts. The APC cohort had an increasing trend of monthly buprenorphine prescriptions prior to the policy changes. After the implementation of the HHS guideline, the APC cohort experienced no change in monthly buprenorphine prescriptions. After the elimination of the X-waiver, the APC cohort continued to experience no change in monthly buprenorphine prescriptions. This result was unexpected as we anticipated that physicians who were primarily writing buprenorphine would transition the prescribing responsibility to APCs on their team after the elimination of the X-waiver, although this may have occurred previously when APCs were granted the ability to prescribe under the Comprehensive Addiction and Recovery Act of 2016. Previous work has shown that among high volume opioid prescribing specialties, APCs experienced the largest increase in buprenorphine prescriptions from 2016 to 2021.^42^ This observed increase in APC prescribing during this time may in part be because of the rapid expansion of the model of addiction medicine clinics after the enactment of the Affordable Healthcare Act and the expansion of Medicaid, as well as telehealth-only MOUD programs. These clinics often rely on APCs to prescribe buprenorphine. More research is needed to understand the flat trend in buprenorphine prescribing among APCs in Massachusetts identified in our study and the current barriers and facilitators for buprenorphine prescribing among APCs as they represent a potential force to increase buprenorphine treatment in the United States.^43^

This analysis demonstrates that the 2 federal policy changes studied in this investigation were not associated with a significant change in the overall trend of total buprenorphine prescribing in Massachusetts. The magnitude of decline in monthly buprenorphine prescriptions among EPs may have been tempered by the elimination of the X-waiver. Additional interventions are necessary to increase prescribing of buprenorphine to patients with OUD.

## Author Contributions

JMH, REC, DDV, ND, LDY, and SGW conceived the study and designed the analysis. DDV, ND, and LDY obtained the data. REC performed the statistical analysis. JMH, REC, and SGW drafted the manuscript, and all authors contributed substantially to its revision. SGW takes responsibility for the paper as a whole.

## Funding And Support

Dr Weiner was supported by 10.13039/100000002National Institutes of Health award 5-R01-DA058315.

## Conflict of Interest

Dr Weiner serves as a consultant for Vertex Pharmaceuticals, Inc, and Cessation Therapeutics, Inc. Drs Hayes, Cash, Ahmad, Menzies, Valerio, Dalvi, and Young have affirmed they have no conflicts of interest to declare.
