# Supplementary material for: The Effect of Federal Policy Changes on Buprenorphine Prescribing in Massachusetts
Source: J Am Coll Emerg Physicians Open. 2025 Sep 9;6(5):100246. doi: 10.1016/j.acepjo.2025.100246 (PMC12454894; doi:10.1016/j.acepjo.2025.100246)
Supplement: Supplemental Appendix 1 [file mmc1.docx]

**Supplementary Appendix 1**. Federal Food and Drug Administration National Drug Codes (NDCs) included in the study

| 00378092493 |
| --- |
| 00228315603 |
| 00228315503 |
| 00228315403 |
| 00228315303 |
| 00093572156 |
| 00093537856 |
| 00054018913 |
| 00054018813 |
| 00054017713 |
| 00054017613 |
| 65162041603 |
| 65162041503 |
| 59385001630 |
| 59385001430 |
| 59385001230 |
| 54123098630 |
| 54123095730 |
| 54123091430 |
| 50383093093 |
| 50383092493 |
| 12496130602 |
| 12496128302 |
| 12496121203 |
| 12496121201 |
| 12496120803 |
| 12496120801 |
| 12496120403 |
| 12496120401 |
| 12496120203 |
| 12496120201 |
